# Versican overexpression as a biomarker in breast cancer diagnosis

**DOI:** 10.1590/1414-431X2026e15306

**Published:** 2026-11-09

**Authors:** G.P.A. da Silva, P.J.C. Ferraz, A.C.S. Ribeiro, R.P. Cavalheiro, M.A.S. Pinhal, C.M. Melo

**Affiliations:** 1 Departamento de Bioquímica, Centro Universitário Faculdade de Medicina do ABC, Santo André, SP, Brasil; 2 Departamento de Biologia Molecular, Universidade Federal de São Paulo, São Paulo, SP, Brasil

**Keywords:** Breast tumor, Chondroitin sulfate proteoglycan, Extracellular matrix

## Abstract

Breast cancer is one of the most lethal malignancies worldwide, with late diagnosis representing a major contributor to its high mortality rate. Consequently, novel biomarkers are being investigated to facilitate earlier detection and better clinical outcomes. Versican, a large chondroitin sulfate proteoglycan found in the extracellular matrix, interacts with diverse proteins and carbohydrates, thereby modulating essential cellular processes such as proliferation, migration, and invasion. These features make versican a promising target in oncological research. In this study, we analyzed versican expression in both surgically resected breast tissue and peripheral blood from breast cancer patients and healthy donors, aiming to evaluate its relevance as a biomarker and its role in tumor progression. To achieve this, we combined *in silico* analyses with *ex vivo* investigations using immunohistochemistry in breast tissues and real-time PCR in the mononuclear fraction. Our findings indicated that versican was significantly upregulated in breast cancer patients compared to healthy controls, in both tissue and blood samples. Taken together, these results underscore the potential of versican as a biomarker for breast cancer.

## Introduction

Breast cancer is one of the most commonly diagnosed cancers worldwide and a leading cause of cancer death in women. One of the main contributing factors to this high mortality rate is late diagnosis (1,2). Breast tumors can be classified into molecular subtypes (luminal A, luminal B, HER2+, and triple-negative (TNBC)) based on the expression of biomarkers such as ER, PR, HER2+, and Ki-67 (3-5). The biological behavior of these subtypes is often influenced by the tumor microenvironment, where specific molecules, such as versican (VCAN), may play distinct roles according to the tumor's molecular profile.

Skandalis et al. (6) analyzed 310 patients with high mammographic density and microcalcifications, findings that are frequently observed in non-palpable carcinomas. Their study revealed a significant increase in *VCAN* expression exclusively in malignant tumors.

VCAN is a large chondroitin sulfate proteoglycan found in the extracellular matrix. Its structure consists of an N-terminal G1 region, which interacts with hyaluronic acid, and a C-terminal G3 region containing epidermal growth factor (EGF)-like motifs. Between these regions, chondroitin sulfate chains are anchored, which can vary in structure and size. At least five isoforms of VCAN are generated by alternative splicing, which may or may not include the central region responsible for anchoring the chondroitin sulfate chains (7,8).

VCAN interacts with various proteins and carbohydrates, modulating essential cellular processes such as proliferation, migration, and invasion. The G1 fraction exhibits anti-adhesive properties, whereas the G3 fraction is associated with the modulation of cell proliferation. These characteristics make VCAN a relevant target in oncological studies (7-9). *VCAN* is expressed in soft tissues, the stroma, tumor, and myeloid cells, being particularly abundant in monocytes. Studies indicate its overexpression in malignant breast lesions, suggesting its potential as a biomarker for breast cancer diagnosis and prognosis (7,8,10).

Despite its known presence in the tumor stroma, it remains unclear whether *VCAN* expression profiles differ significantly across molecular subtypes or if its systemic levels in peripheral blood correlate with tissue expression. This study addresses this gap by investigating the rationale that *VCAN* expression is differentially regulated according to tumor aggressiveness. Thus, we analyzed *VCAN* gene expression in tissues obtained from surgical resection and peripheral blood from breast cancer patients, aiming to investigate its relevance as a biomarker and its role in tumor progression.

## Material and Methods

The study was approved by the Clinical Research Ethics Committee of the Centro Universitário FMABC (Decision number: 58135422.0.0000.0082 - 013/2005).

### Bioinformatics

#### Genomic database analysis

The analysis of genomic databases was conducted at Centro Universitário Faculdade de Medicina do ABC (Santo André, Brazil), using data obtained through the GENT2 software. The *in silico* analysis was performed in February 2025.

#### VCAN expression analysis in tumors

Gene Expression Database of Normal and Tumor Tissues (GENT2) (http://gent2.appex.kr/gent2/) (11) is an online tool that enables gene expression analysis across 72 tissue types, both normal and tumor-derived, based on public databases comprising a total of 68,000 samples.

GENT2 enables the analysis of public transcriptomic data from the National Center for Biotechnology Information (NCBI)-Gene Expression Omnibus (GEO) database, primarily utilizing Affymetrix U133A and U133Plus2 microarrays. In this study, the software was employed to investigate and compare *VCAN* expression in healthy breast tissues (control, n=475) relative to specific breast cancer molecular subtypes (luminal A, n=379; luminal B, n=244; basal, n=363; triple-negative breast cancer (TNBC), n=251; and HER2+, n=230). To assess broader expression patterns, *VCAN* levels were also analyzed in a comprehensive primary breast tumor dataset (n=5,574) *versus* normal clinical samples using the GPL570 platform. Statistical comparisons between groups were performed using the two-sample *t*-test or ANOVA, with significance levels automatically adjusted by the platform to ensure robust differential expression analysis.

### Immunohistochemistry

Breast tumor tissue fragments and adjacent non-tumoral breast tissue fragments (serving as internal controls) were collected from 3 female patients prior to the initiation of systemic treatment. The use of paired samples from the same patient ensures that the genetic background remains constant between tumor and control groups. The specimens were fixed in 10% buffered formalin, processed, and embedded in paraffin.

The paraffin blocks were sectioned into 5-µm-thick slices and subjected to heat-induced epitope retrieval using citrate buffer (pH 6.0). The primary polyclonal anti-versican antibody (Santa Cruz Biotechnology^®^, USA) was diluted in 1X phosphate-buffered saline (PBS) (1:100), supplemented with 1% bovine serum albumin (BSA) and 1% Triton X-100, and incubated for 18 h at 4°C. Following washes with 1X PBS, immunohistochemical staining was performed using a biotinylated secondary antibody conjugated to a peroxidase complex (LSAB+ System-HRP, Dako North America, Inc., USA).

Image analysis was conducted using a Nikon microscope (Japan), and images were quantified with ImageJ software (NIH, USA). To ensure objectivity, the quantification was performed by an investigator blinded to the clinical data and sample identity. Three random fields were selected per tissue section, and 3 histological sections were performed per block, totaling 9 images per sample. Quantifications were conducted at a magnification of 100×, following the protocol described by de Matos et al. (12).

### PCR from peripheral blood

Peripheral blood samples were collected from female breast cancer patients (n=23) and female healthy donors (n=16), all of whom were over 18 years of age. For the patient cohort, longitudinal samples were obtained at 3-month intervals prior to the initiation of systemic treatment. The mononuclear fraction of peripheral blood was purified using Ficoll-Paque (Sigma-Aldrich, USA). Total RNA from the mononuclear fraction was purified using TRIZOL^®^ (ThermoFisher, USA). RNA samples were solubilized in 20 µL of nuclease-free water and quantified using Nanodrop^®^ (ThermoFisher). cDNA synthesis was performed using 400 ng of total RNA with the qPCRBio^®^ kit (PCR Biosystems, USA).

For the analysis of *VCAN* gene expression, quantitative polymerase chain reaction (qPCR) was performed using 100 ng of cDNA and SYBR Green (Life Technologies, USA). The specific primers for *VCAN* were: 5' GCTGCTCAGAAGGCTTGTTT and 5' GACTCCTGCCTTTCCCATCT. The endogenous control genes used for normalization were Beta-actin (*BACT*) and Ribosomal protein L13a (*RPL13a*). The primer sequences were as follows: *BACT*: F: 5′ CCCTGGAGAAGAGCTACGAG 3′; R: 5′ CAATGCCAGGGTACATGGTG 3′; *RPL13a*: F: 5′ CCTGGAGGAGAAGAGGAAAGAGA 3′; R: 5′ TTGAGGACCTCTGTGTATTTGTCAA 3′.

qPCR cycling conditions consisted of an initial denaturation at 95°C for 2 min, followed by 40 cycles of 95°C for 5 s and 60°C for 30 s. The amplification efficiency for each primer pair was validated using a standard curve of serial cDNA dilutions, maintaining values between 90 and 110%.

### Statistical analysis

All statistical analyses were conducted using GraphPad Prism software (USA). Data normality was assessed using the Shapiro-Wilk test. For comparisons between two groups, the unpaired Student's *t*-test was used for parametric data, while the Mann-Whitney and Wilcoxon tests were applied for non-parametric data. For comparisons among multiple groups, one-way analysis of variance (ANOVA) followed by Tukey's *post hoc* test was performed for parametric distributions.

Outliers were identified using the ROUT method (Q=1%) and excluded from the final analysis to ensure data robustness. Additionally, receiver operating characteristic (ROC) curve analysis was performed to evaluate the diagnostic potential of *VCAN* expression. For all statistical tests, a significance threshold of P<0.05 was strictly adopted to define statistical relevance.

## Results

### Expression in breast tissue

Using bioinformatics tools, we demonstrated an increased *VCAN* expression in breast cancer patients (n=5,574) compared to healthy individuals (n=475) (Figure 1). *VCAN* expression was higher in tumor tissue than in healthy breast tissue.

**Figure 1:**
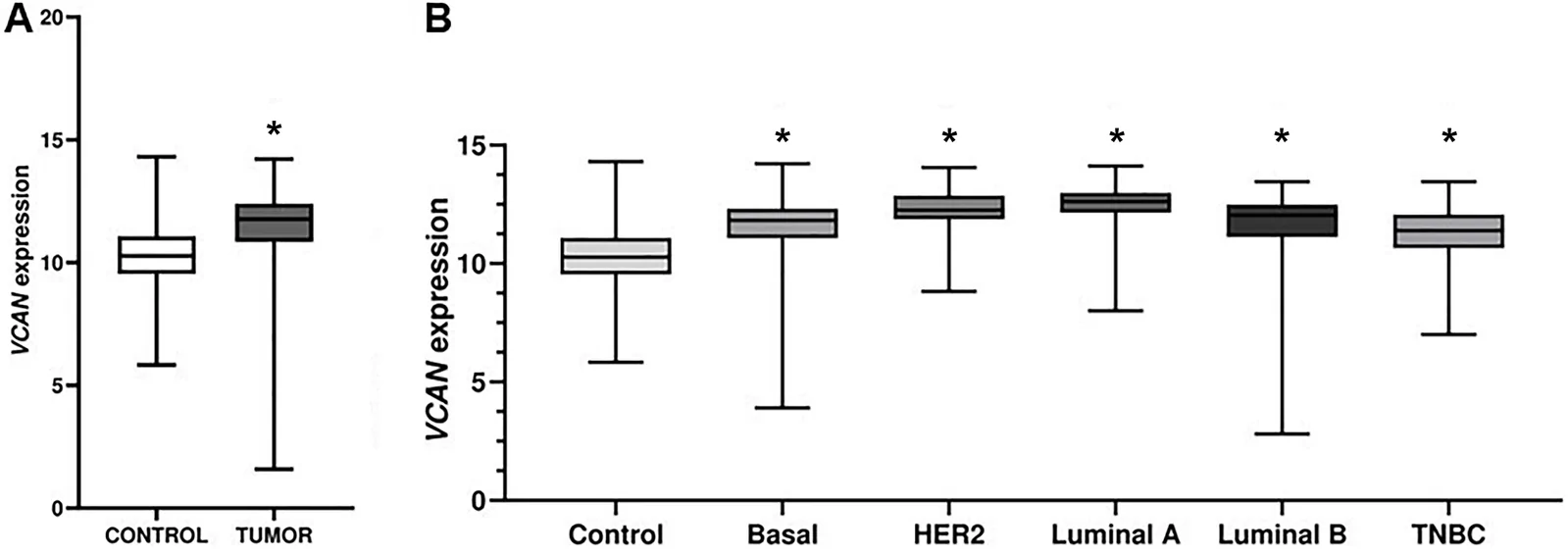
Comparison of versican (*VCAN*) expression in normal tissue and breast cancer tissue in GPL570 extracted from the Gene Expression Database of Normal and Tumor Tissues (GENT2) database. **A**, Control (healthy breast tissue) and tumor (breast cancer tissue). **B**, Control and breast cancer types. Data are reported as medians and interquartile range. *P<0.05 compared to control according to Student’s *t*-test (A) and one-way ANOVA followed by Tukey's *post hoc* test (B).

Breast cancer is a heterogeneous disease, both morphologically and molecularly (2); however, regardless of the tumor subtype, *VCAN* expression was elevated compared to healthy tissues (control n=475) (Figure 1).

To validate the findings from the bioinformatic analyses, we evaluated VCAN protein expression through immunohistochemistry. Quantification of the absorbance demonstrated a significant increase (P<0.05) in VCAN relative expression in breast cancer tissues (143.67±30.01) (n=3) compared to matched normal tissues (113.68±20.63) (n=3) (Figure 2).

**Figure 2:**
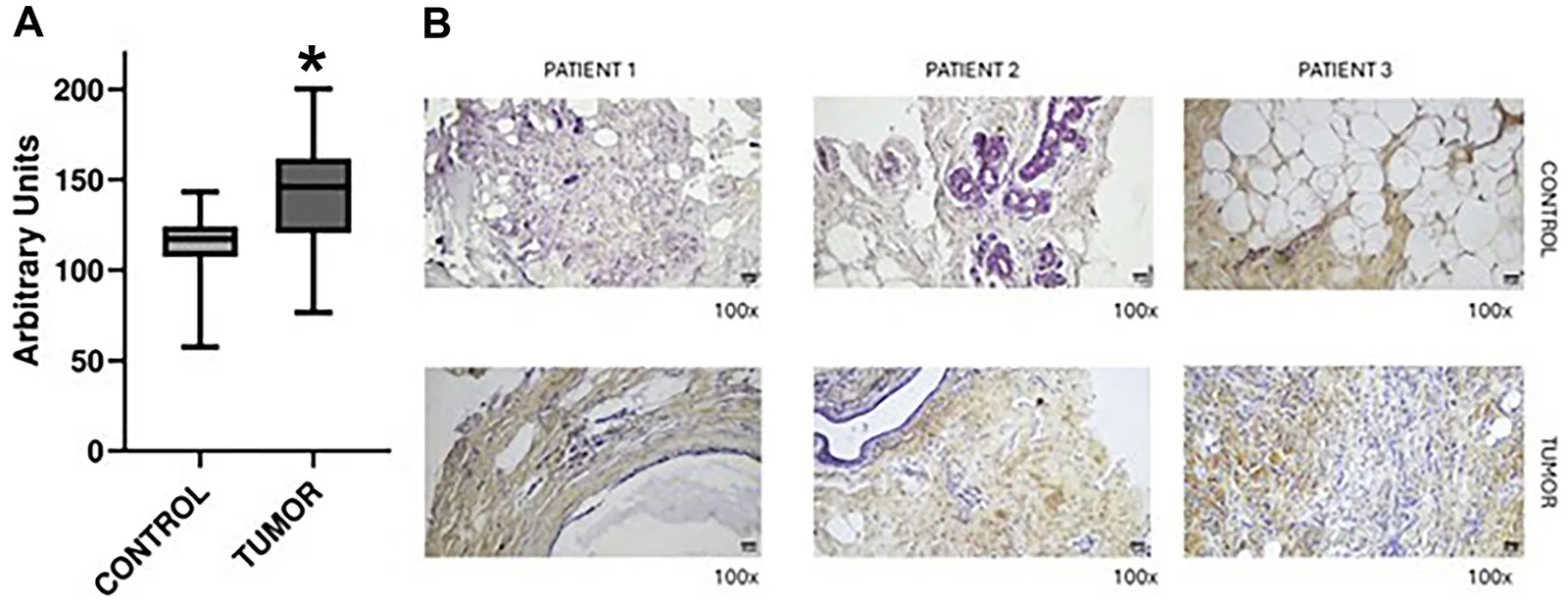
Immunohistochemical analysis. **A**, Immunohistochemical quantifications of versican (VCAN) in tumor tissues and healthy tissues. Data are reported as median and interquartile range. *P<0.05, Wilcoxon test. **B**, Microphotographs of immunohistochemistry analysis. Scale bar=10 µm. In blue, nucleus labeling. In brown, VCAN labeled with polyclonal antibody. To minimize biological variability, tumor tissues and healthy tissues were obtained from the same surgical specimen of each patient (n=3).

To ensure technical and biological consistency, the normal and tumor tissues were obtained from the same surgical specimen of each patient, providing internal controls (matched pairs). The variables considered for this matching required genetic background identical for both samples and hormonal status for both tissues exposed to the same systemic environment.

### PCR from peripheral blood

Breast cancer patients showed a significantly increased *VCAN* expression in the mononuclear fraction of cancer patients (n=23) compared to healthy donors (n=16) (Figure 3).

**Figure 3:**
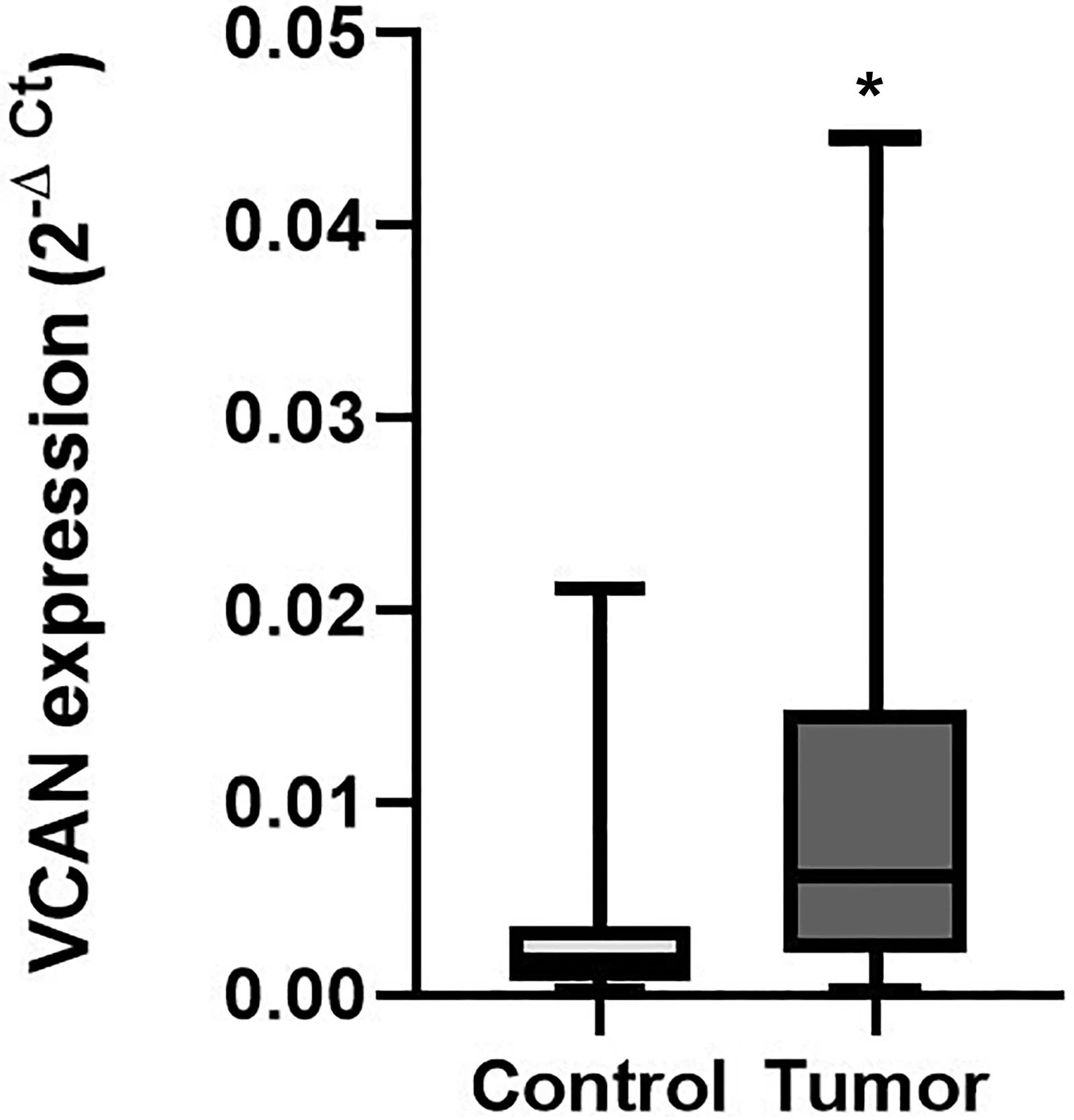
Versican (*VCAN*) expression in the mononuclear fraction obtained by real-time PCR from control (healthy female donors (n=16)) and tumor (breast cancer patients (n=23)) groups. Data are reported as medians and interquartile range. *P<0.05, Student's *t*-test.

The diagnostic performance of *VCAN* was evaluated using ROC curve analysis. The area under the curve (AUC) was 0.78 (95%CI: 0.65-0.899), indicating a good discriminative ability between the tumor and control groups. Based on the Youden Index, the optimal cut-off value was determined to be >0.0048. At this threshold, the assay demonstrated a sensitivity of 56.5% and a high specificity of 93.8%. The positive predictive value (PPV) and negative predictive value (NPV) were 92.9 and 60.0%, respectively (Table 1), suggesting that the marker is particularly effective at minimizing false-positive results in this study population (Figure 4).

**Figure 4:**
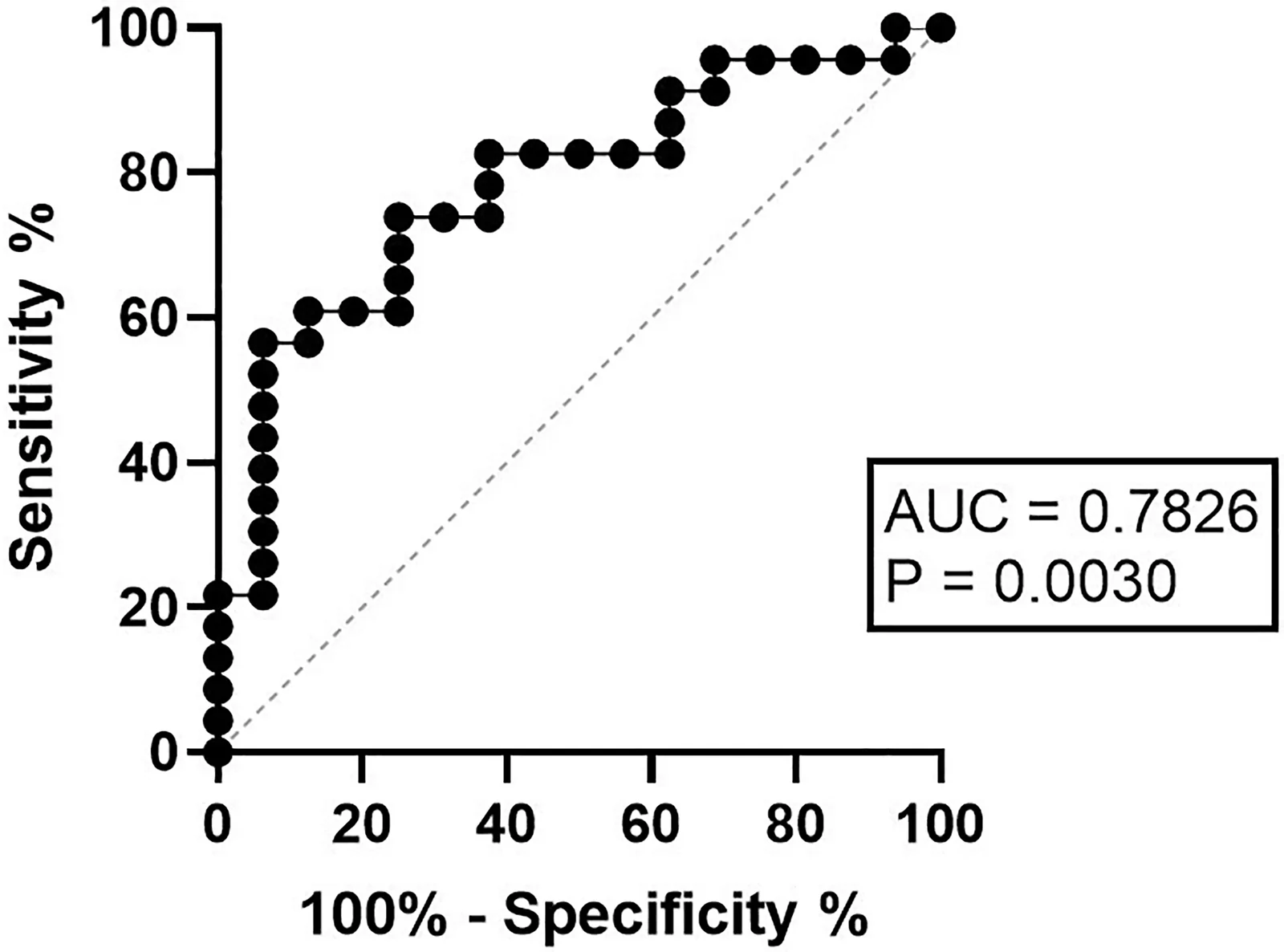
Receiver operating characteristic (ROC) curve of versican (*VCAN)* expression in the mononuclear fraction. AUC: area under the curve.

**Table 1:** Summary of the confusion matrix (at the optimal cut-off point).

	Tumor	Control
Positive test	13 (TP)	1 (FP)
Negative test	10 (FN)	15 (TN)

TP: True positive; FP: False positive; TN: True negative; FN: False negative.

## Discussion

The composition and organization of the extracellular matrix (ECM) in tumors differ significantly from those in healthy tissues, directly impacting tumor growth, invasion, and metastasis. Various ECM molecules play a crucial role in elucidating the molecular mechanisms underlying carcinogenesis and have the potential to be utilized as tumor biomarkers (13). Proteoglycans (PGs) and glycosaminoglycans (GAGs), the major macromolecules of the ECM, are well established in cancer initiation and progression, encompassing the regulation of cellular metabolism, modulation of immune surveillance, and contribution to therapeutic resistance. These macromolecules actively participate in key oncogenic processes, including angiogenesis, inflammation, cell proliferation, and tumor metastasis. Dysregulation of ECM remodeling creates a microenvironment conducive to tumor development by altering cellular interactions and signaling pathways, where ECM components interact with various cytokines and growth factors to influence cancer pathophysiology (14).

In this context, several studies have reported increased *VCAN* expression in malignant tumors, including lung cancer, hepatocellular carcinoma, colon cancer, glioma, and ovarian cancer (7,8,15-17). Specifically in breast cancer, elevated *VCAN* expression has been correlated with a higher risk of recurrence and increased relapse rates in node-negative invasive cases (18), being associated with higher tumor grade, increased invasiveness, and the presence of malignant-appearing microcalcifications (6). Our immunohistochemical analysis demonstrated a markedly elevated expression of VCAN in breast cancer tissues compared to their corresponding normal counterparts, a finding validated by bioinformatic analysis. These results align with the study by Kischel et al. (10), which demonstrated *VCAN* over-expression in breast tumor tissues.

Beyond the tumor site, our study corroborated findings by Theodoro et al. (19), who demonstrated that the mononuclear fraction of peripheral blood from breast cancer patients exhibits an altered expression pattern, suggesting an important function of proteoglycans in crosstalk between circulating lymphocytes and tumor cells. We observed that breast cancer patients showed increased *VCAN* expression in this fraction, similar to what Masuda et al. (20) demonstrated in systemic sclerosis, where *VCAN* is upregulated in the monocyte fraction due to the inflammatory microenvironment, a feature shared by cancer. Additionally, other studies have reported increased VCAN expression in plasma from lung cancer patients, suggesting its potential role as a non-invasive biomarker (17).

While other biomarkers like ctDNA and specific microRNAs are currently being tested in large clinical cohorts for early detection, VCAN stands out for its role in reflecting stromal changes.

Despite the potential of VCAN, its proposal as a diagnostic biomarker requires careful consideration of its biological specificity. Since VCAN is upregulated in several inflammatory conditions, systemic inflammation could act as a confounding factor in its clinical application. Furthermore, the moderate performance observed in our ROC curve analysis indicates that, while promising, VCAN might be more effective when integrated into a multi-marker panel rather than as a standalone diagnostic tool. Compared to established biomarkers like CA 15-3 or emerging ones like ctDNA, VCAN offers a unique perspective on the tumor-stroma interaction, but its moderate sensitivity necessitates further refinement.

The strength of this study includes the multi-level analysis (tissue, blood, and bioinformatics), although it is limited by the sample size. Future studies should focus on validation in larger cohorts and direct comparison with other circulating biomarkers to confirm its clinical relevance and potential for patient monitoring.

## Conclusion

This study advances the understanding of breast cancer pathophysiology by demonstrating that *VCAN* is not only overexpressed in the tumor stroma across various molecular subtypes but is also significantly elevated in the mononuclear fraction of peripheral blood. Taken together, our findings suggest that *VCAN* levels reflect the dynamic remodeling of the extracellular matrix and the systemic inflammatory response associated with malignancy, positioning it as a promising candidate as a non-invasive complementary biomarker.

Future research should focus on validating these results in larger, multicenter cohorts to establish robust clinical cut-off points and to evaluate the prognostic value of *VCAN* in predicting treatment response and disease-free survival. Furthermore, investigating the integration of *VCAN* into multi-marker panels may enhance the sensitivity of current diagnostic strategies for early-stage breast cancer.

## Data Availability

All data generated or analyzed during this study are included in this published article.
